# *'He usually has what we call normal fevers’*: Cultural perspectives on healthy child growth in rural Southeastern Tanzania: An ethnographic enquiry

**DOI:** 10.1371/journal.pone.0222231

**Published:** 2019-09-11

**Authors:** Zaina Mchome, Ajay Bailey, Shrinivas Darak, Flora Kessy, Hinke Haisma

**Affiliations:** 1 Department of Demography, Population Research Centre, Faculty of Spatial Sciences, University of Groningen, Groningen, the Netherlands; 2 National Institute for Medical Research, Mwanza Centre, Mwanza, Tanzania; 3 Department of Human Geography and Spatial Planning, International Development Studies, Utrecht University, Utrecht, the Netherlands; 4 Manipal Academy of Higher Education, Manipal, India; 5 Amrita Clinic, Prayas Health Group, Pune, India; 6 Tanzanian Training Center for International Health, Morogoro, Tanzania; 7 International Union for Nutrition Sciences Task Force ‘Toward Multi-dimensional Indicators of Child Growth and Development, London, United Kingdom; ESIC Medical College & PGIMSR, INDIA

## Abstract

**Introduction:**

While parents’ construction of and actions around child growth are embedded in their cultural framework, the discourse on child growth monitoring (CGM) has been using indicators grounded in the biomedical model. We believe that for CGM to be effective, it should also incorporate other relevant socio-cultural constructs. To contribute to the further development of CGM to ensure that it reflects the local context, we report on the cultural conceptualization of healthy child growth in rural Tanzania. Specifically, we examine how caregivers describe and recognize healthy growth in young children, and the meanings they attach to these cultural markers of healthy growth.

**Methods:**

Caregivers of under-five children, including mothers, fathers, elderly women, and community health workers, were recruited from a rural community in Kilosa District, Southeastern Tanzania. Using an ethnographic approach and the cultural schemas theory, data for the study were collected through 19 focus group discussions, 30 in-depth interviews, and five key informant interviews. Both inductive and deductive approaches were used in the data analysis.

**Results:**

Participants reported using multiple markers for ascertaining healthy growth. These include ‘being *bonge*’ (chubby), ‘being free of illness’, ‘eating well’, ‘growing in height’, as well as ‘having good *kilos*’ (weight). Despite the integration of some biomedical concepts into the local conceptualization of growth, the meanings attached to these concepts are largely rooted in the participants’ cultural framework. For instance, a child’s weight is ascribed to the parents’ adherence to postpartum sex taboos and to the nature of a child’s bones. The study noted conceptual differences between the meanings attached to height from a biomedical and a local perspective. Whereas from a biomedical perspective the height increment is considered an outcome of growth, the participants did not see height as linked to nutrition, and did not believe that they have control over their child’s height.

**Conclusions:**

To provide context-sensitive advice to mothers during CGM appointments, health workers should use a tool that takes into account the mothers’ constructs derived from their cultural framework of healthy growth. The use of this approach should facilitate communication between health professionals and caregivers during CGM activities, increase the uptake and utilization of CGM services, and, eventually, contribute to reduced levels of childhood malnutrition in the community.

## Introduction

Malnutrition is one of the most serious health problems affecting under-five children in Sub-Saharan African (SSA) countries, including Tanzania [1,2]. While malnutrition rates in Tanzania have declined for under-five children since 1999, the rate of chronic malnutrition or stunting remains high, at 35% [1,3]; and exceeds 40% in some parts of the country [1,3,4]. Overall, more than 2.7 million under-five children in the country are stunted [1], a condition that is likely to impair their future learning, productivity, and opportunities to escape poverty [5]. Thus, malnutrition remains the single greatest cause of child mortality in Tanzania [1,6,7]. The prevalence of childhood malnutrition–and of stunting in particular–is higher in rural than in urban communities [1,3,4].

Nutrition initiatives in Tanzania date back to the country’s independence in 1961, at which poverty, disease, and low levels of knowledge–key causes and consequences of malnutrition–were declared major enemies of the nation’s development [6]. However, high-level policy attention to nutrition was witnessed during Millennium Development Goals (MDGs) era, when the Tanzanian government intensified the discussion on nutrition issues in developing its National Strategy for Growth and Reduction of Poverty II (NSGRP II 2010/1011-2014/2015), and launched the National Nutrition Strategy (NNS) 2011/12-2015/16 and its implementation plan. Despite the vast efforts made to address undernutrition in children, the rate of stunting hardly changed during the MDG era [8]. As part of renewed efforts at accelerating and scaling up nutrition action in the country, the government has recently taken important steps, including integrating nutrition into national planning and budgeting, reviewing and updating the 1992 into a 2016 National Food and Nutrition Policy (NFNP), and launching the National Multisectoral Nutrition Action Plan (NMNAP) for the 2016/17-2020/21 period (6). The NMNAP is regarded as a ‘double duty’ multi-sectoral action plan, as it addresses both undernutrition (acute malnutrition and stunting) and the emerging double burden of malnutrition (ibid).

Additionally, the government of Tanzanian, together with various partners, has been implementing recommended preventive and curative interventions, including the early diagnosis and treatment of malaria in children [1,9], and the increased coverage of essential vaccination programs and micronutrient supplementation programs, such as vitamin A supplementation and deworming [6]. Similarly, given that in Tanzania undernutrition manifests at an early age, more emphasis has been placed on routine CGM for under-five children [7,10]. While the nutritional strategic frameworks stress the importance of aligning cultural contexts in designing and implementing interventions targeting childhood malnutrition, the growth monitoring approach used in Tanzania [11], as in other countries [12,13], employ indicators grounded in the biomedical model. Although health workers tailor advice to the specific situation of each individual child, the basic message conveyed to mothers is derived from the biomedical model [14]. The reliance on this model persists even though caregivers’ views are rooted in their cultural framework, which includes ideas about health and growth. A lack of sensitivity to the local constructs and meanings attached to child growth can impair communication between health professionals and caregivers [14,15], thus, the advice provided may fail to have the intended effect [12].

Previous research on child growth in Tanzania focused on determinants of childhood malnutrition, which is reasonable given the high prevalence of undernutrition in young children and its major contribution to child morbidity and mortality. For example, poor growth has been found to be more prevalent in the children of women in polygamous marriages [16,17], particularly the children of first and second wives [16]. Substantial farm labor demands placed on the mothers [17,18], the household’s characteristics, and the family’s economic status [19] have been identified as significant risk factors for stunting in under-five children. An ethnographic study among the Hadza noted that the degree of a child’s relatedness to the male head of the household mediated the quality of care the child received [20]. Similarly, certain aspects of Chagga history, demography, political economy, and culture have been shown to contribute to the inability of families to provide adequate childcare [21]. The evidence presented above indicates that household and societal arrangements influence caregivers’ (and particularly mothers’) ability to provide the care needed to ensure that their children are growing well. These findings do not, however, explain how caregivers conceptualize ideal child growth. By understanding caregivers’ perceptions of healthy growth, healthcare providers will be in a better position to encourage growth and discourage malnutrition in children.

Although the Tanzanian government and other health programmers have tried to make child growth-related interventions accessible to most communities, little is known about how health service providers can collaborate most effectively with caregivers in improving child growth. This study aims to fill that gap by using cognitive anthropological perspectives of the cultural meaning system in exploring ‘emic’ perspectives on healthy child growth. We seek to answer two main questions. First, how do caregivers describe a child who is growing well? Second, what meanings are attached to these cultural markers of growth? Only by understanding the constructs and meanings caregivers assign to healthy child growth can public health professionals (PHPs) become culturally competent enough to develop culturally-embedded interventions [22]. In health programming for the treatment of acute respiratory infections, the identification of symptoms as perceived by the mothers in their respective socio-cultural contexts helped PHPs design messages that motivated mothers to seek medical care for their sick children [23]. Similarly, a recent systematic review on the sustainability of health interventions in SSA found that the integration of community values into the development and implementation of interventions increased the likelihood of their sustainability [24]. Designing interventions that incorporate local constructs around healthy growth will not only facilitate nurse-caregiver communication and increase the utilization of services; it will increase caregivers’ sense of ownership and involvement. Thus, the use of this approach increases the chances that interventions will be sustainable, and will prove successful in reducing child malnutrition.

According to D'Andrade [25], a community’s cultural meaning system is composed of shared cultural schemas. These cultural schemas form the reality-defining system and provide information about what states of the world can be and should be pursued (ibid). These schemas are, therefore, context-specific interpretive devices (ibid) that facilitate the creation of knowledge and the attribution of meaning to objects [26]. Thus, how a certain health condition is conceptualized (e.g., healthy child growth) depends on the cultural meaning systems of each community (ibid). Straus and Quinn [27] have argued that schemas exist for all kinds of phenomena (including child growth), and are embedded in broader systems of cultural meaning. While some schemas are individual to a person, others, cultural schemas, in particular–are shared by a group of people. Such schemas are highly internalized, and are thus difficult to change [28]. The wider community’s cultural schemas inform individuals’ beliefs and perceptions, and shape their behavior and experiences, including those related to child growth. The cultural schemas theory was relevant to this study, as it helped the researchers understand how caregivers conceptualize healthy child growth in their local setting, and the meanings they ascribe to markers of healthy growth.

## Methods

### Study setting

The ethnography was conducted in a rural setting of Kilosa District, Southeastern Tanzania. Kilosa is predominantly rural, and agriculture is the backbone of its economy. In collaboration with district leaders, one village was selected for this study based on its rural location and its access to CGM services. The availability of CGM services in this village enabled us to capture caregivers’ perceptions of these services and their interactions with health workers. Like other parts of Kilosa district, the study village is a multi-ethnic settlement, with fertile land and weather conditions conducive to agriculture that attract internal migrants. There are two main ethnic groups in the study village. The first is the native Bantu group, to which the majority of the population belong. The Luguru, Sagara, Kaguru, and Pogoro people are in this group. Other minority Bantu ethnic sub-groups that have migrated to and settled in this area include the Sukuma, Nyamwezi, Zaramo, Gogo, and Hehe people. The second group is the Maasai, a Nilotic minority group who have migrated to this area in the past two decades in search of pasture for their livestock.

Most of the people living in the area are subsistence farmers who cultivate rice and maize, particularly during the rainy season that usually runs from November to June. During the dry season, some of the population attempt small-scale irrigation gardening. While most of the residents are engaged in agricultural activities, the Maasai people are mainly focused on livestock keeping, with a few households cultivating maize and rice for food.

Like many other rural villages in Kilosa District, the village is poorly served in terms of health infrastructure. As there are no health facilities in the village, the residents depend on health services provided in other villages outside of the ward that are approximately 3–6 kilometers away. The road to these villages is poor, and is passable only during the dry season. Within the village, there are two small privately owned *maduka ya dawa* (drug shops), both of which are operated by unqualified attendants who offer a range of medications mostly without prescription, including antibiotics and antimalarials.

### Study design and methods

The study employed an ethnographic approach to examine caregivers’ constructs and meanings they attach to healthy child growth. As Field & Morse [29] has observed, the knowledge gained through ethnography is ‘emic’ in nature, which conforms to our objective of understanding healthy child growth from the local people’s perspective. The fieldwork was undertaken for three months in the study setting using multiple data collection techniques, including focus group discussions (FGDs), in-depth interviews (IDIs), key informant interviews, and field notes (see Table 1).

**Table 1:** Information for FGDs, IDIs, & KIIs participants.

Activity	Number of times the activity was performed	Age range	Gender		Level of education	Total number of participants
			Male	Female		
FGDs	19	18–74	39	98	0 –form IV	137
IDIs	30	17–71	11	19	0 –form IV	30
KIIs	5	39–50	1	4	0 –standard 7	5

### Participant recruitment

The participants in the FGDs and the IDIs were (1) mothers and fathers who had under-five children, regardless of their nutritional status; and (2) women aged 45 years and older. Community health workers (CHWs) and traditional birth attendants (TBAs) were interviewed as key informants. The purposive sampling technique was used to recruit the participants. For the FGDs, older women and parents of under-five children were identified from the general community with support from local leaders and influential people. Many of the IDI participants were identified with the help of the CHW and through the researchers’ social networks. The key informants were selected based on their experience with and knowledge of the issues of research interest. The recruitment of the participants continued up to the point of data saturation, when no new information related to the topics of interest was obtained from the interviews.

Two researchers participated in the data collection process: the principal researcher (first author) and a trained Tanzanian female research assistant with a postgraduate social science backgrounds in sociology. Both researchers had advanced training in qualitative methods and extensive experience in qualitative data collection procedures. The researchers’ access to the study community was facilitated by a number of factors, including their proficiency in Swahili (the language spoken by almost all of the local people), as well as their skills in establishing rapport. The principal investigator (first author of this paper) was pregnant during the first round of interviews, and took her newborn daughter with her in the field during the second round, which made it easier for her to establish rapport with the mothers and their children. Being a mother of an under-five child also gave her the opportunity to attend growth monitoring clinics, where she could observe the mothers’ activities and interactions around growth monitoring issues.

### Data collection

The data presented in this article are part of a larger ethnographic study on the socio-cultural dimensions and contexts that underlie growth of under-five children in Southeastern Tanzania. Prior to the start of the actual fieldwork, the data collection instruments were pilot tested. The results from the pilot study helped the team refine the guides before they were used in the main study. The ethnography (actual field work) was conducted in two phases. From July to September 2015, the researchers conducted 19 focus group discussions (seven with mothers, five with fathers, and seven with elderly women). These activities facilitated the researchers’ access to the community, and enabled them to capture the general perceptions of child growth in the community and the general contexts underlying growth of under-five children. Before continuing with the next phase of data collection, we transcribed and coded all FGD recordings. Our aim was to be able to adapt the interview guides based on the FGDs.—something which enabled us to validate the information generated through the FGDs, and to address the gaps that were identified. From August to September 2016, 30 in-depth interviews (12 with mothers, 11 with fathers, seven with older women) and five key informant interviews with CHWs and traditional birth attendants were conducted. The interviews captured the caregivers’ personal views on healthy child growth, and information on ways in which they were tracking growth in their individual children. Topic guides with open-ended questions were used in both the interviews and the FGDs, the duration of which ranged from 50 to 90 minutes. The IDI guides were adopted before the start of the second round to address the gaps in the information obtained through FGDs. Emic perspectives on healthy child growth (a focus of this paper) were mostly captured under the topic ‘community/parents’ views and opinions on (ideal) child growth’. In the FGDs, a general question was posed: ‘In your community, how can someone know that a child is growing well?’ The participants were probed on a range of markers used in ascertaining healthy growth in a child. During the IDIs, parents were asked for their views on the growth of their individual under-five children. The parents who reported that their children were growing well were asked to justify their statements. In line with local conceptualization of healthy growth, the participants were also asked for their opinion on CGM activities (Please see S1–S6 Files). All of the interviews and FGDs were recorded using digital recorders, and were transcribed verbatim. The FGDs and interviews were conducted in Swahili, which is the national language in Tanzania. Swahili is spoken by all of the people in the community, and is the mother tongue of the PI. The interviews and FGDs were conducted in locations chosen by the participants. While most of interviews were conducted in the participants’ homes, the FGDs took place in different venues in the village, such as school classrooms (after school hours), and the principal researcher’s and the participants’ home compounds. Other than the participants and the researchers, no one else was present during the FGDs and IDIs.

### Data analysis

To preserve linguistic authenticity [30], all of the transcripts were analyzed in the original language; i.e., in Swahili. Following the completion of the data transcription, the first author read all of the transcripts to verify that they accurately represented the audio files. She then read the transcripts line by line to identify initial categories and crosscheck the analytical concepts that emerged during the fieldwork. The analysis took place at two levels. At the first level, the inductive and deductive codes were developed. At the second level, the patterns and relationships between the categories were identified and the main themes were synthesized, which reflected the cultural conceptualizations and meanings attached to healthy child growth. Some themes represented new concepts that emerged inductively from the data, while other themes reflected the theoretical components that informed the data collection topic guides. After the deductive and the inductive concepts were combined, the following themes emerged: (i) being *bonge*, (ii) being free of illness, (iii) eating well, (iii) growing in height, (iii) having good *kilos*, and (iv) contradictions between cultural and biomedical assessments of healthy growth. NVivo 11 software (QSR International Pty Ltd, Australia) was used to facilitate the data analysis. Field notes were used to clarify and expand the caregivers’ meanings by providing additional data not included in the transcripts. In the process of writing this paper, the research team jointly discussed which quotes would effectively illustrate the themes identified throughout the process of analysis.

### Ethical issues

The study was approved by both the University of Groningen Research Ethics Committee and the Tanzania Ministry of Health, Community Development, Gender, Elderly and Children (MoHCDGEC) through the Medical Research Coordinating Committee (MRCC) of the National Institute for Medical Research (NIMR). All interviews and FGDs were conducted after receiving written/thumbprint consent from each participant. Permission to conduct this study was also sought from regional, district, and community leadership prior to the commencement of any research activity. In addition, the researchers conducted community sensitization meetings and which they introduced themselves and the purpose of the study to the community members. Although local leaders were used in approaching some of participants, the recruitment process ensured that each participant was informed of the research and his/her right to voluntary participation in the study prior to his/her participation. All interviews and FGDs were conducted after receiving written/thumbprint consent from each participant. The confidentiality and anonymity of the study participants was assured by using nicknames to identify them during the fieldwork. Any real names of participants that were captured accidentally in the recordings of conversations during FGDs or interviews were removed during the data-cleaning process. Thus, the names used in this article are not real names. The study did not pose any risk to participants.

## Results

The participants mentioned multiple gauges and specific markers that they have used to ascertain healthy growth in a child. In this section, we present five markers (see Table 2) that the community members reported using to identify healthy growth in a child, and the meanings they attach to each marker. We start with those markers that reflect cultural constructions, followed by those that contain both cultural and biomedical elements. We also highlight the contradictions between cultural and biomedical markers of healthy growth. In the quotes from FGDs and IDIs, ‘I’ stands for ‘interviewer’ and ‘P’ stands for ‘participant’. Although it was not the goal of this study to compare the participants based on ethnicity, we note that our analysis found no cultural differences in perspectives on healthy child growth between the ethnic groups represented in the study setting.

**Table 2:** Healthy growth markers as identified by the study participants and biomedical indicators.

Indicators	Emic markers	Biomedical markers
Being *bonge* (chubbiness)	- Becomes fat - Body expands - Is looking fat - Has a good body - Looks healthy - Clothes are tightening - Growing out of clothes - Waist chain tightens - Traditional bracelet tightens - Fleshy round cheeks - Fleshy body	Body composition
Being free of illness	- No prolonged illness - Not intermittently sick - Malaria fever passes away from the child - Going a long time without malaria - No malaria since birth -Has ordinary malaria fever • Malaria easily cured • Malaria but no weakness • Malaria but no hospitalization • Malaria fever calmed down by Panadol • Malaria that quickly responds to treatment • Malaria but no weight loss	Healthy
Eating well	- Eats every food given - Is not choosy - Finishes usual portion of food - Demands food when hungry - Eats according to stomach size - Stops eating when full	Has good appetite
Growing in height	- Looking taller than before - Clothes become too short - Height increases - Takes things from high places the child could not reach before - Shoots up	Height for age
Having good kilos (Weight)	- Is heavy (when carried) - Feeling overwhelmed / tired when carrying the child for a long distance - Weight marked in green area of growth chart - Weight gain accelerates - Has kilos - Kilos do not go down	- Weight for age - Weight gain - Weight marker in green area of growth chart

### Being *bonge* (chubby)

The participants reported using a child’s appearance to ascertain healthy growth, saying they believe a child who is growing well is chubby or has a large body. As one key informant put it: *‘A healthy and well growing child has a big body size’* (CHW, 50yrs, KII). Remarks such as *ananenepa* (‘is becoming fat,)’ *ni bonge* (‘is chubby’), *ana afya* (‘is healthy),’ or *ana mwili* (‘has a good body’) were commonly used to describe a child who was growing well. A child’s body size was also cited by some parents of under-five children as one of the first signs they look for in judging a child’s growth at birth:

As soon as the baby is born… the first thing that you do when you enter the room to see the baby is to check her body. *Hajakondeana*? (‘Is s/he not skinny?’). Is s/he fat? Thus, you look at her and say, ‘Yes! I have got a perfect *mama*’ (referring to a daughter) (a father, FGDs).

The caregivers–and particularly the women–reported using their child’s clothing size as a gauge of growth, observing that a child is considered to be growing if his/her clothes seem to be tightening over time:

You can tell by looking at her clothes. When you see that some of them are tightening, you then know she is growing (a mother, FGDs).

Some of the older women added that an increase in a child’s body fat could be ascertained by looking at the traditional bracelet (*kamwa*) around the child’s hand or waist. If the bracelet tightens, it is clear that the child’s body has grown:

The measures we use at home include clothes and black threads (traditional hand/waist bracelets). When the thread starts to tighten, you know the child is growing. So you expand the size. When it tightens, you know the child is moving (growing). You then say, ‘This one is grown up now’ (an older woman, FGDs).

While the majority participants ascribed a child’s fatness to good nutrition or genetics, some of the older women said they believe that a child’s fatness is a function of God’s will (*Mpango wa Mungu*), and some perceived it as a negative effect of childhood vaccinations. Although several participants said they cherish a chubby child, they also noted that not all fatness indicates healthy growth, and differentiated between ‘good’ and ‘bad’ fatness. Bad fatness was believed to be caused by and indicate diseases in a child. A child with bad fatness was described as one who looks fat, but is less active and light, like a floating buoy (‘*mnene na mwepesi kama boya’*).

R: I would like to clarify; there are two sorts of fatness. You may find that a particular child is fat but has some problems, even his weight is not sufficient. But another child may be fat and her/his health is good. He grows well.
I: What is the one who is fat and grows well like?
R: S/he is fat and active (*unene wake wa kuchangamka*). Just by looking at her/him you have to say that this one is good. But for the one who is ‘stunted’ (general term for poor growth), you find that s/he is fat but her/his fatness is different. S/he is not as active as his/her fellow who has healthy fatness (CHW, 40 yrs, KII).

The interpretations of and meanings attached to fatness in a child expressed by the participants were rooted in the community’s cultural meaning system regarding body image, and were specifically influenced by the cultural value that chubbiness connotes good health and beauty. The community’s system of assigning meanings to body image appeared to inform the mothers’ schemas for their own children and those of others, with many saying they envy the parents of children who look chubby and have fleshy cheeks:

P: A fat child is more beautiful than a thin one. I can see what the children of other people look like.
I: Why do you say that she is beautiful?
P: Everyone praises her, saying ‘This child is *bonge* (chubby). She is indeed *bonge*.’ As a mother, that makes you very satisfied. You are satisfied that your child is fat. She is in good health (a mother, 40 years, IDI, farmer).

When your neighbor gives birth to a baby with a good body, one whose face looks nourished, and her little cheeks are fleshy, you say, ‘Kha! My neighbor has given birth to a beautiful baby. What a pleasure!’ That is what we say; that her baby is growing well. When you carry her, you feel that today you have carried a beautiful baby. I can feel her weight in my hands (a mother, FGDs).

Similarly, in the participants’ cultural context, chubbiness in a child is seen as an indicator that the parents are providing good care. Thus, when a child looks fat, the parents are praised by community members as being good caregivers:

When someone’s child is chubby, people say, ‘Ah, our colleague has done a great job. She takes good care of her child’ (an older woman, FGDs).

Labeling chubby children as ‘healthy’ or ‘beautiful’ could lead to the social exclusion and stigmatization of children whose bodies are small. Several of the participants spontaneously reported that community members enjoy carrying a chubby child and are less interested in carrying a thin one. In describing the stigma thin children face, parents in different FGDs recounted that people in the community have gossiped about the health of a particular thin child:

A child who is fat is very much cherished by people in the community. In most cases, people say, ‘Ah, look at that child of [name], she looks attractive’. That child is very much loved by many people in the community. Even when she crawls toward someone’s feet, the person feels happy and picks her up. But if a child seems thin, people may say, ‘Ah, look at that little one, I think s/he has some problems’ (a father, FGDs).

### Being free of illness

Whether a child is free of illness, and particularly of malaria, is also seen as an important dimension of healthy growth. In their accounts, participants used the term *‘homa’* (fever) interchangeably with ‘*maleria*’, a local term for malaria. The participants describeddelay in seeking treatment for an ill child. farm activities health workers also cause the importance of attending GM clinics g a child who is growing well as resistant to illness, using phrases such as ‘fevers / illnesses pass away from her’, ‘she does not get fever frequently’, s/he ‘is not frequently ill’, and s/he ‘has ordinary fever/malaria’ (‘*homa / malaria ya kawaida’*). The caregivers said they can tell whether a child has malaria by touching his/her skin for warmth or by taking him/her to the hospital for diagnosis. A child’s resistance to illness, and to malaria in particular, was spontaneously mentioned as an important indicator that a child is growing well:

When malaria passes away from her, then I know that my child is growing well. So, that is the indicator [*kiashiria*] of a child whose growth is healthy (a father, FGDs).

A child who is growing well does not get sick frequently. He gets sick very rarely (CHW, 50 years, KII).

I: How do you see the growth of your grandchild [name]?
R: Janet is growing well. I know that she grows well as ‘her fevers are the normal ones’ (*homa zake ni za kawaida*). So, she grows well. When she has a fever (malaria) and you give her medicine, she recovers easily (*anapona tu kirahisi*). Unlike others who can get the dosage but are resistant to it (the medicine)! It is not until s/he is admitted that her fever calms down. But for her, when she has fever (malaria) and you give her ‘*mseto’* (ALu), she gets well. We in the villages do not have further expertise. When you look at the child and see that s/he does not get fever frequently, we see that s/he is growing well (TBA, 48 yrs, KII).

In the in-depth interviews, most of the parents reported that their child was growing well because she had *homa ya kawaida* (‘ordinary fever’)–referring to malaria fever that ‘does not weaken the child or make her thin’, ‘is easily treated or cured with pain relievers or home remedies’, and ‘does not lead to hospitalization’. Having an ‘ordinary fever’ was thus seen as a sign that the child was growing well. In this case, self-medication and the delayed seeking of treatment for a child’s illness was described as common:

John’s growth is normal. He usually has what we call normal fevers. When his fever comes, it does not necessarily require you to buy ‘*mseto*’ (ALu) or any other anti-malaria drug. You only buy him Panadol and the fever comes down. We then say that the fever has perished (a father, 48 years, IDI, farmer).

Zuena is growing well. She does not have frequent fever. Her fevers are ordinary. When she has a fever, I take her to the hospital. She is given medicine and goes up to six months, eight months, or even one year before getting malaria again (a mother, 42 years, IDI, farmer).

In the study community, the high rate of infectious diseases, and particularly of malaria, appears to have permeated people’s cultural meaning systems, leading them to conceptualize healthy growth as being based on a child’s ability to fight off *homa* (malaria). Demonstrating how community members’ shared experiences with malaria infection have led them to have similar schemas, one of fathers in the FGDs explained that the community members’ perceptions of fever have been shaped by the pervasiveness of malaria in their region:

Where we live, a child who is growing well is one who is not often ill. This is because in our environment, diseases such as malaria tend to attack our children and retard their growth. Even if a child was growing well, her growth may falter if she gets malaria. Thus, you are very lucky if your baby has never had a bad case of malaria; you conclude that your child is indeed growing well (a father, FGDs).

### Eating well

A child’s eating habits emerged as one of the dimensions that community members use to discern healthy growth in a child. Many of the participants described a child who is growing well as one who eats or breastfeeds well. A child is considered to be eating well if s/he has good appetite, never vomits after eating, is not picky about the food s/he eats, finishes his/her usual portion, stops eating when full, and demands food when s/he feels hungry:

A child who is growing well is one who likes to eat. Even if s/he is still breastfeeding, if you give her/him porridge s/he drinks it. When you give her *ugali* (stiff porridge) s/he as well eats. His/her growth must be good as s/he likes to eat and s/he breastfeeds well (TBA, 48 yrs, KII).

Each child has a specific amount of food that s/he can eat. The one who does not grow well eats too much. He eats beyond his belly capacity. But the one who grows well eats moderately; s/he does not eat too much. His/her self becomes satisfied (*nafsi yake inaridhika*). S/he is not greedy; s/he stops eating when s/he is full. [laughter] (CHW, 50 yrs, KII).

In line with the widely held belief in her community that ‘good eating’ indicates healthy growth, one mother said she thinks her child has been growing well because he eats every food she prepares for him:

Jackson’s growth is good. He eats well. He never chooses what to eat. He is not picky. He eats everything that I prepare for him. He never hesitates to eat; he eats everything normally. He eats everything put in front of him (a mother, 42 years, IDI, farmer).

The parents of under-five children said they have expectations about the amount of food each of their children can finish, as they have studied their children’s eating behavior every day. Some reported serving food to each child on a separate plate to track how much each child has been eating. Being able to finish the food provided was characterized as a good sign of growth. When asked how she could be sure her child was eating well, one of the mothers replied:

I can see myself that my child is eating well. As I feed him, I can see that he eats well. There is a particular cup that I use; it is quarter-liter of porridge. When I give him that one, he drinks all of it. If I add some porridge and give it to him, he refuses. Then I know that my child is full. So I know his portion. If he is eating his favorite food and I realize he is eating much more than the usual amount, I stop him. I know that he is continuing to eat out of greediness (a mother, 36 years, IDI, farmer).

The participants’ use of the child’s eating habits as a dimension of healthy growth was embedded in their schemas regarding illness as a cause of a child’s lack of appetite. Many of the parents explained that when their child eats less food than expected, it alerts them to the possibility that the child is developing an illness, particularly malaria or intestinal worms.

I know my child Mohamed very well. When I buy him a plate of *chipsi mayai* (French fries mixed with eggs), he usually finishes it. If he eats only a small portion of it or eats less than usual, I become worried that he is getting sick. It may be intestinal worms or malaria (a father, 54 years, IDI, farmer).

### Growing in height

Although the community members were aware of their child’s height, most did not talk about height when conceptualizing healthy growth. Nevertheless, a child’s stature was described as an important sign by some of the caregivers. During the interviews, a few of the parents said they determine whether their child has a healthy rate of growth by looking at whether his/her height has been increasing steadily:

Hawa is growing well. I can see that she is growing tall, while some children (in the same cohort) are not getting taller (a father, 50 years, IDI, farmer).

The lack of a concept of height in the framework of growth described by most participants seemed to emanate from their beliefs about the origin of their child’s height, with many saying they feel they have no control over their child’s stature. Although some of the participants indicated they are aware that having a good diet has a positive impact on growth, most said they believe a child’s stature is mainly determined by God’s will or heredity (more details presented in [31].).

### Having ‘good kilos’ (weight)

Most of the participants reported using weight to ascertain healthy growth in a child. They described a child who is growing well as heavy *(‘ana kilo’*) or as gaining weight as she grows. Weight was also used to judge a child’s growth at birth. Parents reported feeling pleased if their child’s birth weight was 3kg or more.

A child grows by gaining weight … If she was born weighing 3kg and later gains up to 10kg or more, you know the child is growing (a father, FGDs).

When asked how they could tell if their child was gaining enough weight, many parents replied that they could sense the child’s weight when carrying or picking him/her up: *‘*We use our hands to know the weight’. Tiredness when carrying a child was the main local marker caregivers used to recognize heaviness or weight gain in their child:

Tausi is growing well. Her weight is increasing nowadays. Even before going for measurements (weighing at growth monitoring clinics), I can tell. She likes to go with us to the farm. In the past, I could carry her to the farm on my shoulders without getting tired. But now, when I carry her I feel like I have high blood pressure (a rapid heartbeat). She is a bit heavier. Her weight has increased (a father, 43 years, IDI, farmer).

When I lift him, he is heavy. If you carried him from here to Kimamba and back, the whole of your chest would be aching. He is heavy, unlike his older brother Omary. I could carry him to Ludewa and come back without feeling he was heavy (a mother, 22 years, IDI, farmer).

Although the participants related good child weight to good nutrition (*lishe bora*), many of the community members, and particularly the older women and the parents of under-five children, said they believe that a child’s weight is not always an outcome of nutrition. In their cultural context, a child’s weight is ascribed to the nature of the child’s bones (*mifupa*). Thus, the participants appeared to believe that some children have bones that are naturally heavy while others have bones that are light, and that the weight of the child’s bones is a function of heredity and God’s will. The observation that twins can have different weights at birth, or that some thin children turn out to have better *kilos* than some who are fat, were among the reasons participants cited to justify their shared schema on a child’s bones. Based on this schema, some participants indicated that they believe that no matter how well a caregiver feeds a child, it is unlikely that s/he will become heavy if his/her bones are light.

The weight of a child emanates from her/his bones. You may have two children, one is thin and the other one fat, but the thin one may have much better *kilos* than the fat one. That is because of her/his bones. Even if you feed her/him with good food, s/he does not become heavy (an older woman, female FGD).

Based on their cultural schema regarding a child’s bones, the participants explained that when a thin child appears to be unexpectedly heavy or when caregivers are unable to find a reason for poor weight gain in a child, the child’s bones are considered to be the source of the problem:

She eats as usual. Why do her *kilos* decrease? S/he has no frequent illness, but her *kilos* decrease. In our rural context, we say this is because of the child’s light bones […] (a mother, FGDs).

The community members’ interpretations of and meanings attached to weight as a dimension of healthy growth were also rooted in their cultural template regarding the etiology of poor weight in breastfeeding children. Some of the participants, mostly women, attributed low weight in a child to the parents’ violation of postpartum sex taboos, including having sexual intercourse while the baby was still breastfeeding or becoming pregnant while the mother was still lactating (*‘kukatikiza’*). Many of the participants said they believe a mother’s breast milk can spoil if she is exposed to a man’s semen or becomes pregnant while lactating, and that the breastfeeding child might therefore contract diarrhea and lose weight. Similarly, having sexual intercourse or becoming pregnant is believed to generate body heat (*joto la mwili*) or sweat (*jasho*) that can have a negative effect on the growth of the child (more details are presented in another manuscript). Based on these shared cultural schemas, picking up a child by holding her/his hands was one of the main methods the community members said they use to confirm that the parents have been adhering to postpartum sex prohibitions:

After giving birth, we do not see men until the child stops breastfeeding. That is a period of two years. He should not touch you, as the sperm and body warmth from sex affect the child. Usually, an elderly person who lives in the neighborhood comes by and says, ‘My in-law, are you up? Please let me carry the baby’. She takes the baby and swings her up and down. You may think she is only playing with the baby, but she is actually checking the weight. If the baby is heavy, she says, ‘Congratulations my daughter, you are indeed nurturing your baby’. If the baby is weak, she asks you, ‘What are you doing to this child?’ (an older woman, age unknown, retired farmer)

In addition to the cultural meanings attached to a child’s weight, the participants demonstrated knowledge of growth in line with the biomedical model. Many reported getting information about their child’s weight through growth monitoring clinics. Unlike the fathers and the older women, the mothers of under-five children and the key informants (i.e., the community health workers and the TBAs) demonstrated some comprehension of the growth chart (clinic card). In ascertaining healthy growth, some of the participants said they pay attention to the monthly weight gain as recorded by the health worker. Most of the participants referred to the color of the area where the child’s weight is marked on the growth chart, with a mark in the green-shaded area indicating healthy growth, and a mark in the gray-or red-shaded area indicating poor growth:

You find that a particular child is seven and a half kilos this month, and when you measure her/him again next month, you find that s/he is eight kilos. That shows that her/his weight has increased. You then realize that this child is growing well (CHW, 40 yrs, KII).

You can tell if a child is growing well. For instance, when we go to the (CGM) clinic and measure her/him, you can see. There are three colors: there is a gray color, a green color and a red color. So if you see that s/he is in a green color, you know that this one is in good health. But if you find him/her in a gray color, then you know that her/his growth is not good. There are three colors; the red one is the most dangerous one. It indicates that this one is not in good health (TBA, 48 yrs, KII).

I can easily see if a child is growing well as I measure them during CGM clinics. You find that a child goes to the green color. S/he never drops into the gray or the red color. Thus, that vividly shows that the growth of that child is going well (CHW, 50 yrs, KII).

However, the participants did not seem to understand the grading of the colors in the growth chart, as they did not refer to the gray color as an indication of obesity, but instead said it signified the onset of weight loss. They also described the red color as reflecting the critical stage of weight loss. The participants’ individual schemas of the color in the growth chart as a marker of child growth was influenced by their experiences with CGM services:

When your child reaches the gray color, it indicates danger, as it shows that her/his *kilos* are starting to go down. If the child drops further and reaches this color (pointing to the red-shaded area of her child’s growth chart), it shows that the child has *utapiamlo* (malnutrition). That is what the red color indicates. That is why they tell us to be vigilant about the two colors (i.e., gray and red) (a mother, 22 years, IDI, farmer).

### Contradictions between cultural and biomedical assessments of healthy growth

Despite the caregivers’ knowledge and use of the CGM weighing services, they seemed to rely on local markers in interpreting the growth of their child. Based on their cultural constructs of healthy growth, they could guess their child’s growth status prior to attending the CGM clinics, and thus used the outcome of the weighing procedure to confirm their initial hunch. Some of the caregivers said that when the CGM results contradicted their expectations, they felt frustrated. For instance, one mother of an under-five child who said she believed that her child was growing well based on a number of cultural makers of healthy growth recalled being confused by the results when she took her child to the CGM clinic:

My child had a good body, she was never ill. Her body was initially small, but she then became fat. So I was pretty sure when I took her to the clinic today that her *kilos* (weight) had increased. But when she was measured, my child was found to have lost weight. That confused me, I felt bad […] (a mother, FGDs).

Although the caregivers expressed positive attitudes towards CGM, their reliance on cultural markers in tracking their child’s healthy growth seemed to mediate their uptake and utilization of CGM services, particularly after the child had completed his/her vaccinations, usually at 18 months. As one community health worker during KII noted: ‘When a child completes the vaccinations (*chanjo*), many stop attending clinics; they perceive it as no longer meaningful’. When asked why they had missed some of their child’s CGM scheduled appointments or stop attending clinics before their child reached age five, the mothers gave responses such as ‘*nilidharau tu’* (I just ignored it), ‘*ni uvivu tu’* (It is just laziness), ‘*sina sababu ya msingi*’ (I do not have any good reason). When probed further, the mothers typically explained their behavior with statements such as ‘I see that my child is doing well’, ‘s/he plays well’, ‘s/he eats well’, ‘I see that s/he is healthy’, or ‘s/he is not sick’ (*haumwi*). All of these reasons reflect cultural markers of healthy growth:

P: I was taking him to [the clinic] for weighing (*kupima*) until he turned one and a half years old. When a child is one and a half years old, there is a certain injection (vaccine). Those (vaccinations) are very important, as they are the *kinga* (prevention against diseases). When we reach that time (18 months), most of us despise the clinic (*tunaidharau*). Sometimes you go, sometimes not.
I: Why did you stop taking your child to the clinic after completing the vaccinations?
P: I just despised it because I see that my child is playing perfectly well. […]. She is okay and is not sick (*Yuko safi haumwi*) (a mother, 36 years, IDI, farmer).

The above shows how the broader systems of cultural meaning inform caregivers’ perceptions about the growth of their child and influence their behavior and decisions in relation to their child’s growth.

## Discussion

The aim of this study was to explore caregivers’ local conceptualizations of healthy growth, and to examine the meanings they attach to the markers used in identifying healthy growth in a child. Understanding the meanings and schemas that underlie caregivers’ conceptualizations of healthy growth could be useful for the improvement of CGM and other childcare services, as the beneficial schemas can help health professionals develop strategies for promoting healthy growth, while the harmful schemas can alert them to issues that may call for intervention [32].

### Medicalization of cultural schemas of healthy growth

The study results show that the caregivers ascertained healthy growth in their children using multiple indicators, and that in applying each of these indicators, they referred to specific local markers. While much of their knowledge of and schemas regarding healthy growth were embedded in their broader systems of cultural meaning, the study participants also integrated ideas derived from biomedicine into their local knowledge and practices. They appeared to be aware of several biomedical signs used to assess growth in young children, such as weight gain and the colors in the child’s growth chart. However, both red and gray colors were interpreted as indicating ‘low weight’. Since the 1970s, Tanzania has been integrating into its pediatric care growth monitoring practices based on WHO’s growth charts. Thus, it is not surprising that the caregivers in our study appeared to have some knowledge of the biomedical markers of child growth. However, although the caregivers used biomedical concepts in their local conceptualization of growth, the meanings they attached to these concepts were largely embedded in their cultural framework. For instance, whether a child had a poor or a good weight was ascribed to the nature of the child’s bones and the parents’ adherence to postpartum sex taboos. The belief that the parents’ non-adherence to post-partum abstinence causes weight loss in infants has also been reported in other qualitative studies conducted in Tanzania [33]. The schema that a child’s weight is a function of his/her bones can cause caregivers to fail to recognize the risk of overweight or underweight in their child, and thus to avoid seeking help. To ensure that caregivers are paying attention to their child’s weight, health workers should educate mothers during CGM clinics that a child’s weight is mainly a nutritional issue. Furthermore, improving caregivers’ comprehension of the message in the growth curve in relation to the growth of their children is important, as it can influence their behavior and decisions related to healthy child growth.

### Body fat and child growth

The cultural norms regarding ideal body size shaped the participants’ conceptualization of healthy child growth, and the participants referred to a child who was growing well as *bonge* (chubby). In most of their accounts of healthy growth, they used the word ‘health’ (*afya*) interchangeably with the word ‘fat’. When asked to describe a child who was growing well, the participants spontaneously said ‘s/he is healthy’ (*ana afya*); which means the child ‘is chubby’. In their context, a fat child was considered beautiful, and fatness was described as an indicator of good health and a higher level of parenting competence. Fatness was also used to ascertain healthy growth at birth. The idea that fatness is a sign of attractiveness, affluence, and good health has also been reported from other settings [32,34]. The study participants’ use of a big body size as a marker of healthy growth at birth could be encouraging given the higher probability of survival of large babies than of small babies. In Tanzania, for instance, more than 80% of neonatal deaths occur among children with low birth weight [8]; and children who are small at birth are more likely to become stunted than those described as being average or large [1]. Nevertheless, the cultural preferences for a large body size in children should be viewed cautiously, as such beliefs can encourage child feeding practices that can lead to childhood obesity, which is particularly relevant given that Tanzania is currently experiencing a double burden of malnutrition [6]. Moreover, as Quinn [35] and Bailey et al. [32] have observed, cultural schemas are a necessary part of individual actions, but they are not always beneficial. For example, the negative stereotypes about children with a small body size found in this study represent harmful schemas that, if left unaddressed, can not only cause distress and stigma for caregivers with thin children, but can cause them to pressure their children to eat [36] and to provide their infants with developmentally inappropriate nutrition, including the early introduction of solids and/or table foods [37–39]. Our findings have implications for programs directed at reducing levels of childhood obesity, as defined by WHO. In order to be effective, programs targeting childhood obesity need to be aligned with cultural interpretations of fatness in a child. Similarly, the integration of the concept of obesity into the awareness messages to mothers may lead them to adjust their perceptions of a large body size in a child.

### Freedom from illness: A gauge of healthy growth

In this study, the participants commonly referred to illness, and particularly malaria, when ascribing meanings to their cultural markers of healthy growth. The pervasiveness of this association may have been influenced by the country’s stage in the epidemiological transition, and the high prevalence of malaria infection in Morogoro [1], the region where this study was conducted. In Tanzania, malaria remains the leading cause of morbidity and mortality for under-five children and pregnant women [1,9,40]. A child’s resistance to malaria infection was spontaneously mentioned as an important marker that led caregivers to assume that the child was growing well. If their child went for long periods of time without a malaria infection, the parents saw this as a sign of good growth. The finding that healthy growth was often tracked using a child’s resistance to infections is an important message. It indicates that poor child growth can be addressed by raising awareness of how to prevent infections, and by encouraging parents to seek treatment for their child if s/he becomes sick.

The majority of parents who participated in the in-depth interviews said they believe their child is growing well if s/he usually has an ordinary fever/malaria [*homa/malaria ya kawaida*]. The cultural naming of ‘febrile illness’ or ‘uncomplicated malaria’ as *homa/malaria ya kawaida* has also been reported by previous ethnographic studies and other studies conducted in Tanzania (cf. [41–45]. However, its use as an emic marker of healthy child growth has not been mentioned previously. The interpretation of mild illness as an ‘ordinary fever’ has important implications for treatment [46]. In this study, for example, the meanings attached to *homa ya kawaida* as a marker of healthy growth seemed to contribute to the caregivers’ tendency to delay seeking help; and to the widespread use of self-medication, particularly painkillers (Panadol), as an initial treatment for a fever. These findings can partly explain the current low uptake of treatment at health facilities among rural children with fever (1), and can be related to the results of other studies in Tanzania. It has, for instance, been shown that in Tanzania, a majority mothers delay taking their child to dispensaries after the onset of a fever based on the belief that their child has *homa/malaria ya kawaida* [42], and instead rely on self-medication [41,42,47,48]. However, in other parts of Morogoro region, the failure to seek prompt and appropriate malaria treatment for children has also been ascribed to issues of affordability, the absence of trusted medical professionals, the unavailability of diagnostic instruments, long waiting times, and long distances [49]. Our study findings suggest that for Tanzania to achieve the global and country targets of reducing the prevalence of child stunting from the current 34% to 28% by 2021, reducing malaria deaths by 80.0% from 2012 levels [9], and achieving Sustainable Development Goal 2 and 3 (improving nutrition and ensuring healthy lives for all), greater community awareness about the importance of the timely diagnosis of malaria and the effective biomedical treatment of the disease is needed.

### Conceptual differences between growth and height

The present study revealed conceptual differences between the biomedical model and the participants’ perceptions of a child’s height. While the biomedical perspective considers height to be an outcome of growth, the participants indicated that they believe that height is not related to nutrition, and that they have no control over their child’s height. The differences in the concepts regarding growth and height in the accounts of the local people in this study and in the biomedical model are consistent with those found in previous research conducted in Guatemala [22] and in rural Mexico [15]. The lack of a concept of height within the framework of growth of most of the participants of the current study seems to emanate from their cultural schemas on the origin of height, as they appear to view a child’s stature as the result of God’s will *(Mpango wa Mungu*) or as function of heredity (For more information, see [31]. This schema is indicative of a failure to recognize nutritional stunting among the caregivers in the study setting, which is of great concern given the high prevalence of childhood stunting in the country [1,3]. As a strategy for improving caregivers’ ability to recognize height deficits as manifestations of malnutrition, Turnbull [15] has recommended integrating the concept of stunted (linear) growth into the awareness messages. While we agree with this suggestion, we would argue that it may take a long time for an emphasis on height to positively affect caregivers’ mindsets, given that the cultural schemas and meanings are shared, created, and performed within the wider community [32], and are thus difficult to change [28]. Basing on our findings, we suggest that Public Health Professionals (PHPs) could be more effective in improving linear growth among children by building upon beneficial schemas held by caregivers. For example, based on the cultural knowledge that a child’s resistance to illness and ability to eat well indicate healthy growth, the PHPs could develop messages that will help them give culturally embedded advice to caregivers about good child feeding practices, about approaches for dealing with their child’s lack of appetite, and about the need to seek early diagnosis and biomedical treatment when a child becomes ill.

### A blend of biomedical and cultural knowledge

The participants appear to have adapted to different sources of available information in making sense of their children’s growth. In line with the biomedical model, they reported paying attention to their child’s body weight, as they seem to believe that being heavier (*kilos*) is an indicator of good growth and good care by the parents, including of the parents’ sexual abstinence during the postpartum period. They also judged whether a child was growing well by applying cultural markers, including whether the child was chubby, had good eating habits, and was free from illness. In this case, the weighing of their baby was seen as simply providing reassurance that all was well. Thus, when the CGM results contradicted their earlier expectations, the caregivers reported feeling frustrated and worried about the growth of their child. Concerns among mothers about their baby’s weight fluctuations have also been reported in other ethnographic studies [39]. The findings of this study further show that after their child had received the important vaccinations, the caregivers tended to abandon the CGM clinics, and resorted to using their cultural markers to ascertain whether their child was displaying healthy growth. This tendency is of great concern, given that in Tanzania, many child health care services are channeled through GM clinics.

The results of this study suggest that the communities in which CGM services are implemented, are not ‘empty vessels’ [50]; but have relevant knowledge about and skills in tracking the growth of their children based on their cultural framework. Therefore, when appropriate, it is important to give caregivers explanations that make sense to them based on their unique frame of reference. The observed tendency of caregivers to stop going to CGM clinics after the vaccination period, and to instead rely on cultural markers of healthy growth, highlights the danger of being blind to the cultural context when developing local interventions. As Hahn and Inborn [50] have cautioned, ignoring the cultural context may lead recipients to reject the PHP’s advice, either because they do not understand it, or because they give it a relatively low priority. To optimize the communication between caregivers and health workers during the CGM clinics, we would argue, along with Turnbull [15], that PHPs should demonstrate cultural competence by using growth monitoring tools that acknowledge caregivers’ constructs of child growth, while complementing them with anthropometric indicators. The recognition of caregivers’ constructs of child growth may create a sense of ownership and improve caregivers’ uptake of CGM services, which could in turn encourage the utilization of important services that can help children achieve optimal growth.

## Conclusions

The study was conducted in a single village, and the data were collected from a small group of participants. Thus, our sample size was not large enough to allow us to make conclusive statements that go beyond the research setting. Even so, because this study was conducted in a multi-ethnic setting, it is possible that the perspectives expressed by the study participants on healthy child growth are similar to those held elsewhere in the country, or even beyond the borders of Tanzania.

This study has shown that caregivers’ conceptualizations of healthy growth are largely rooted in their cultural meaning systems. Accordingly, for health workers to provide advice to mothers during CGM appointments that is sensitive to their specific context, a tool that takes into account the mothers’ constructs of healthy growth is needed. The lack of a concept of height in the participants’ framework of healthy growth is of great concern given the high prevalence of childhood stunting in the country. PHPs should use the study’s findings to take advantage of the beneficial schemas held by caregivers to promote linear growth, rather than simply height. The cultural construction of ‘uncomplicated malaria’ as a gauge of healthy child growth reflects the normalization of this life-threatening illness, which may hinder prompt malaria treatment and contribute to the low utilization of biomedical treatment for children who are ill. To improve the effectiveness of large-scale malaria control programs in Tanzania, and to encourage healthy child growth, PHPs should pay closer attention to the socio-cultural knowledge and meanings attached to symptoms of malaria [42,43].

## Supporting information

S1 File FGD guide for fathers of under-five children. (DOCX)

S2 File FGD guide for mothers of under-five children. (DOCX)

S3 File FGD guide for older women. (DOCX)

S4 File IDI guide for fathers of under-five children. (DOCX)

S5 File IDI guide for mothers of under-five children. (DOCX)

S6 File Topic guide for key informant interview. (DOCX)
