# Supplementary material for: 'He usually has what we call normal fevers’: Cultural perspectives on healthy child growth in rural Southeastern Tanzania: An ethnographic enquiry
Source: PLoS One. 2019 Sep 11;14(9):e0222231. doi: 10.1371/journal.pone.0222231 (PMC6738644; doi:10.1371/journal.pone.0222231)
Supplement: S3 File — (DOCX) [file pone.0222231.s003.docx]

**Appendix 2b: Guide Questions for FGD Sessions (Grandmothers of Under-five children)**

I would like to thank you all for coming today. My name is …………… and my colleague is (mention the name). As earlier informed, we will conduct focus group discussion with various people in this community as part of activities in my PhD study on issues related to growth of young children and monitoring of child growth. We believe that through talking to you about your views and opinions on growth of children will help the researcher to learn a number of issues on how growth is understood in this community. Let me inform you the modality of our group discussion today. As explained earlier, your participation to this study is voluntary so you are free to decide whether to participate or not. We however value your views and hope that you will participate. During the discussion, please be free to express your views as there is no right or wrong question. It is highly insisted that everyone respect freedom of expression of other participants. It is also very important that only one person talks at a time so that all information shared during this discussion is well recorded.

During the discussion, my colleague (name) will take notes and support me in asking questions whenever necessary. I would like request for your permission to use a small recording machine to record the conversation during the discussion. Therefore, we request that you speak aloud so that what you say is recorded clearly. In order to avoid interruptions to the recording we would like to request that you put your mobile phones in silent mode or switch off during the discussion. Our discussion will last about an hour or so. At the end of this discussion, we will provide you with a bottle of a soft drink. Are there any questions before we start?

**A: Participants’ background information**

|  | Age | Sex | Tribe | Religion | Education level | Occupation | Marital status | Number of children | Age of youngest |
| --- | --- | --- | --- | --- | --- | --- | --- | --- | --- |
| 1 |  |  |  |  |  |  |  |  |  |
| 2 |  |  |  |  |  |  |  |  |  |
| 3 |  |  |  |  |  |  |  |  |  |
| 4 |  |  |  |  |  |  |  |  |  |
| 5 |  |  |  |  |  |  |  |  |  |
| 6 |  |  |  |  |  |  |  |  |  |
| 7 |  |  |  |  |  |  |  |  |  |
| 8 |  |  |  |  |  |  |  |  |  |

**B: Introduction Questions**

Let us start by a brief introduction. Please introduce yourself and tell us who you are and type of work you do.

-Can you describe the main income generating activities for men in this village? How about those of men?

- Please tell us about types of foodstuffs that are available in this village?

-How do you get these foods?

-Can you describe the main type of food that people in your village like to eat?

1. When people talk about ‘a young child’ in your community, what are they referring to? (If not mentioned, probe on other aspects than age).
2. In this community, who is considered to be a good mother to her young children?
3. What do people in this community see as the roles of a grandmother for a child to grow well?)

**C: Community views and opinions on ‘ideal’ child growth**

1. When a child is born parents expect to see their child grows well. In your community how is a child expected to grow from when it was born to age 5? If not mentioned, probe this with reference to sex of a child (Ask about the past and now)
2. How can someone know that a child is growing well? (Probe on a range of criteria used in assessing healthy growth). Probe on healthy growth at birth, at age 1, 2, and 5 year old). (In the past and now)
3. Children are routinely taken to health facilities for growth monitoring. Does this happen in your village? Can you explain what things are checked for by health workers when assessing growth of children? What does it mean to you?
4. For many years now in child care programs growth of young children has been assessed by measuring their weight for age. What do you think about this? What other issues would you suggest to be considered in assessing growth of young children? (For each response, ask why?)
5. What do people in your community think about height of a child? Does it have any association with good or bad health? (Probe on: Interpretations of short stature in children and determinants of height of a child; probe on difference between ‘normal short stature and ‘kudumaa’ i.e. stunting). (probe about the past and now)
6. Some of the children appear to be too fat than others. What is your opinion on a growth of a child who is fat? (probe about the past and now).
7. Some of the children have low weight compared others of the same age. What are your opinions on a child who has low weight than others of the same age (probe what determines the weight of a child)
8. In this research we realized that there are practices that are conducted to facilitate healthy growth of a child since when it is born. Please tell me what are practices that parents in this community do to promote growth of their children (Ask the participants to narrate the practices from when a child is born up to age 5). (probe about the past and now)

**D: Community Perceptions on growth faltering**

1. In your community, how can a poorly growing child be described? (probe about the past and now)
2. How can someone recognize that there is a problem in the growth of a child? (Probe on criteria used in identifying poor growth in children). (ask about the past and now)
3. Majority of under-five children in the country are reported to suffer from growth problems. In your experience, what might be the factors that contribute to poor growth of young children in your community (Probe on all perceived causes of poor growth including poor income, kubemenda, single motherhood, mother’s lack of time, infections, beliefs, food issues, environmental & socio-cultural limitations, etc.) (ask about the past and now)
4. Mothers are key caregivers of under-five children. In this research we realized that some of caregivers face some challenges that hinder their ability to provide good care to their children. In your experience, what might be possible factors that challenge the ability of mothers in your village to provide good care to their young children? (Probe on poor income, single motherhood, mother’s lack of time, infections, beliefs, food issues, environmental & socio-cultural limitations, etc). (ask about the past and now)
5. What do parents / caregivers in your community usually do when they think that there is a problem in growth of their children? (Probe: for different actions that caregivers take and reasons for that e.g. go to health facilities, consulting traditional healers, use traditional herbs, -what motivates their decisions?). (ask about the past and now)

-What are specific health / growth issues in children that necessitate the decisions to consult traditional healer or use of traditional medicine?

1. What are traditional preventive measures that people in your community take against growth problems in their children? (Probe from when a child is born up to age five (For each practice probe for the reasons). (ask about the past and now)
2. What do you think should be done to make sure that children grow well? (ask about the past and now)

**E: Feeding Practices**

1. Kindly tell me about child feeding practices in your community. (Probe on the following:

- Timing of introduction of breast feeding

-Timing of introducing complementary feeding (type of complementary food). Reasons for introducing complementary food before six months)

-When do mothers wean their children? (Weaning food, Reasons for weaning a child before reaching two years of age)

-Type of food that is commonly fed to young children in her family (ask the participants to specify foods for various categories of under-fives)

-How many times children are fed in a day, quantity, (ask this for various categories of under-five?)

-Eating habits e.g. whether children and adults eat together in one bowl etc.

-Preparation methods and storage of foods

1. We are now reaching the end of our discussion. Are there any other comments about children’s growth that you want to make before we conclude?

**Thank you for your time and participation to this study.**
