# Supplementary material for: 'He usually has what we call normal fevers’: Cultural perspectives on healthy child growth in rural Southeastern Tanzania: An ethnographic enquiry
Source: PLoS One. 2019 Sep 11;14(9):e0222231. doi: 10.1371/journal.pone.0222231 (PMC6738644; doi:10.1371/journal.pone.0222231)
Supplement: S4 File — (DOCX) [file pone.0222231.s004.docx]

**Appendix 3b: Topic guide for IDI – Fathers of under-five children**

**IDI Code number: ………………………..**

**Date: …………………………**

**Participants’ background information**

I would like to ask you some questions that will help me know more about you.

| Age | | Tribe | | Religion | | Educ level | Can read | Occupation | Marital status | No of children | Living with mother of child/ren? | Number of adults living with you? | Relationship? |
| --- | --- | --- | --- | --- | --- | --- | --- | --- | --- | --- | --- | --- | --- |
|  | |  | |  | | Father …………………….  Partner ……………………. | Yes  No | Father  Partner | Single  Monogamous marriage  Polygamous marriage  Cohabiting  Separated  Divorced  widower |  | Yes  No |  |  |
|  | **Details about participant’s under-five children** | | | | | | | | | | | | |
| Name of a child | | | Gender | | Date of birth | Relationship | Still alive? | Age at last birthday? | If dead, age when s/he died. | If dead, perceived cause of child’s death. | | | |
| ----------- | | | Boy  Girl | | Month ……  Year….. |  | Yes  No | Year |  |  | | | |
| ---------- | | | Boy  Girl | | Month ……  Year….. |  | Yes  No | Year |  |  | | | |
| ---------- | | | Boy  Girl | | Month ……  Year….. |  | Yes  No | Year |  |  | | | |
| ---------- | | | Boy  Girl | | Month ……  Year….. |  | Yes  No | Year |  |  | | | |
| ----------- | | | Boy  Girl | | Month ……  Year….. |  | Yes  No | Year |  |  | | | |
| ---------- | | | Boy  Girl | | Month ……  Year….. |  | Yes  No | Year |  |  | | | |

**Opening questions**

Would you please tell me something about your daily activities? (Probe on: main source(s) of family income).

What does taking care of a child entail? (Probe: purchasing food, cooking for a child, feeding, bathing, putting a child to bed, setting a child up for pooping, cleaning a child after pooping, wash child’s clothes, take a child to health care, education etc…)

How do you describe a young child? (age, not knowing things, helpless, does not know what is good and bad for him/her, preschool)

**Perceptions of Child growth**

1. How does a healthy child look like? How about the one who is not healthy? (Probe on markers).
2. What do you think about the health of [*name*]? Is she/he healthy? Why do you say so? (Probe for markers)
3. Please tell me about the growth of [*name*]? Is she/he growing well? If yes, how do you know that [*Name*] is growing well? [If not mentioned, probe: social interaction, weight, activeness, playfulness, eating habits, sleeping habits, emotional, cognitive ability, motor development, recognizing people / things, body size / being fat, height (Check for gender differences in each aspect).

-Ask for her perceptions of child’s height (probe for her Interpretations of short stature in a child, and determinants of height of a child; difference between ‘short stature and *kudumaa*: probe for markers of *kudumaa*)

-Probe for her opinion on the growth of a child who is fat (what makes a child to be fat?)

-Probe on her perception of child’s weight in relation to her/his growth (determinants of weight of a child?)

-Probe: What do you do to make [name] grow well? (Probe: traditional preventive measures, sleeping under a bed net, environment is clean, take to hospital for check-up, nutritious food etc). What more do you wish you could do?

-In case [Name] was mentioned to not grow well, ask: why do you think so? Please tell me about the growth experience of [Name]. (Probe on markers i.e. physical appearance, social interaction, weakness, not standing, inactive, not speaking, skin, hair, cheeks).

-What do you think made [Name] to not grow well?

-What did you do when you realized that there is growth issue in [*Name*]? (Probe for different actions e.g. go to health facilities, consulting traditional healers, use traditional herbs, -Ask what motivates her decisions?)

(Please ask about growth scenario that was not the case in QN 3). Ask: do you know of a child that grew well? / did not grow well? Please tell me about her/him. How did he/she look like? What do you think contributed to poor growth of that child?

1. Are you aware of ‘utapiamlo’? How does a child with ‘utapiamlo’ looks like? (Probe for markers); probe for his opinions on causes and remedies of ‘utapiamlo’ in children.

**Contexts of child growth**

1. As a father, what are your responsibilities within the family? How does that influence growth of your children?
2. Who takes care of [Name]? Who else helps her with taking care of [name] when she is (a) at home but busy (b) away from home for attending her daily responsibilities (c) the child is ill (d) the caregiver is ill? - In each response probe for type of support provided by each person).

-Probe: How do you support a mother of [name] in taking care of her/him? (Probe on types of support e.g. taking children to growth monitoring, hospitals, paying for school fees, feeding children: Check for direct care that a father provides to [Name]; and the ones that a father has never provided to [name]. Why?

-Probe for the role of his (a) mother and his (b) mother in-law in care provision to his child (staying with a child, advice on child feeding, decision making on child care, caring mothers during pregnancy, advice on child’s illness & treatments, a help in initiating and ending breastfeeding, etc)

1. What is your opinion on the care that your wife / primary caregiver of your child provide to him/her? (Probe: At some points, have you ever been not happy with the care that your wife/caregiver provides to your child? Please tell me about that.

-Probe: please tell me about the environment that you think shape the quality of care that she provides to your [name] (probe for favorable and unfavorable factors)

-As a father, what more support do you think you need to provide to your wife to make [Name] grow well?

1. As a father, please tell me something about the environments that mediate your efforts to provide good care to [Name]. (Probe on: wife’s / partner’s behavior, relationship with his wife or child’s mother, access to resources, decision making on resources, family income & child care; division of labor, child’s behaviors, environment, health system, food issues, community factors, income, health issues, socio-cultural norms over foods, beliefs, socio-cultural practices, marital status i.e. single, polygamous married etc).

-Can you think of a time that you wanted to take care of your child, but you could not? Please tell me about that?)

1. As a father, what support do you need from your wife/partner, family, community, environment, health workers, policy makers) to make [Name] grow well?
2. What do you think of ‘kubemenda’? Has it ever happened to a child of any of people you know? Please tell me a story about it. (Probe on implication to couple’s relationship, community’s attitude? Who is generally blamed, in what way? What do you think about it?]

-Probe for: his opinion about growth of the baby who (a) is conceived while a mother is still breastfeeding (b) growth of unborn child whose parents continue having sex.

1. Do you know of a person that stays with a child who is not her/his own? What are the reasons that made that person to stay with the child? How do you consider the growth of that child? Why do you say so? (Probe on environment that the participant thinks influences growth of that child).
2. In this village, where do you get water for domestic use? (probe on multiple water sources, main and regular water source for drinking, How they prepare water for drinking, child feces disposal, place for hand-washing, use bush/field for open defecation, reliability and seasonality of water sources, time to collect water during wet and dry seasons).

**Experience with growth monitoring services**

1. What do you do when you realize that [N*ame*] is ill? (Probe for different actions eg. go to health facilities, consulting traditional healers, use traditional herbs, -Ask what motivates his decisions? Check for availability of health facility in the area - check who is involved in child health care decisions?
2. Please tell me about growth monitoring services offered in your area (probe on a place where it is offered, who is providing the service, how often, if he takes his child to growth monitoring)
3. Can you show me your youngest child's growth chart? What was your child’s weight at birth? How do you understand the chart? (colors, position in the chart) What do you think about this? (Probe on participant’s opinion on other criteria to be considered in assessing growth of under-five children? Note if both weight and height have been measured.
4. We are now approaching end of our discussion. Do you have anything more to add on your child’s growth and the environment of her/his care?
