# Supplementary material for: 'He usually has what we call normal fevers’: Cultural perspectives on healthy child growth in rural Southeastern Tanzania: An ethnographic enquiry
Source: PLoS One. 2019 Sep 11;14(9):e0222231. doi: 10.1371/journal.pone.0222231 (PMC6738644; doi:10.1371/journal.pone.0222231)
Supplement: S5 File — (DOCX) [file pone.0222231.s005.docx]

**Appendix 3a: Topic guide for IDI – Mothers of under-five children**

IDI Code number: …………………..

Date: …………………………………..

**Participant´s background information**

I would like to ask you some questions that will help me know more about you.

| Age | Tribe | | Religion | | Educ level | Can you read? | | Occupation | Marital status | No. of children | Living with father of child/ren? | Number of adults living with you? | Relationship with adults? |
| --- | --- | --- | --- | --- | --- | --- | --- | --- | --- | --- | --- | --- | --- |
|  |  | |  | | Mother  Partner | Yes  No | | Mother……………..  Partner…………….. | Single  Monogamous marriage  Polygamous marriage  Cohabiting  Separated  Divorced  Widow |  | Yes  No |  |  |
|  | **Details about participant’s under-five children** | | | | | | | | | | | | |
| Name of a child | | Gender | | Date of birth | Relationship | | Still alive? | Age at last birthday? | If dead, age when s/he died. | If dead, perceived cause of child’s death. | | | |
| ----------- | | Boy  Girl | | Month ……  Year….. |  | | Yes  No | Year |  |  | | | |
| ---------- | | Boy  Girl | | Month ……  Year….. |  | | Yes  No | Year |  |  | | | |
| ---------- | | Boy  Girl | | Month ……  Year….. |  | | Yes  No | Year |  |  | | | |
| ---------- | | Boy  Girl | | Month ……  Year….. |  | | Yes  No | Year |  |  | | | |
| ----------- | | Boy  Girl | | Month ……  Year….. |  | | Yes  No | Year |  |  | | | |
| ---------- | | Boy  Girl | | Month ……  Year….. |  | | Yes  No | Year |  |  | | | |

**Opening questions**

Would you please tell me something about your daily activities? (Probe on: main source(s)of family income)

What does taking care of a child entail? (Probe: Cooking for a child, feeding, bathing, putting a child to bed, setting a child up for pooping, cleaning a child after pooping, wash child’s clothes, take a child to health care, education …)

How do you describe a child? (age, not knowing things, helpless, does not know what is good and bad for him/her, preschool)

**Perceptions of Child growth**

1. How does a healthy child look like? (Probe on markers). How about the one who is not healthy?
2. What do you think about the health of [*name*]? Is she/he healthy? Why do you say so? (Probe for markers)
3. Please tell me about the growth of [*name*]? Is she/he growing well? If yes, how do you know that [*Name*] is growing well? [If not mentioned, probe: social interaction, weight, activeness, playfulness, eating habits, sleeping habits, emotional, cognitive ability, motor development, recognizing people / things, body size / being fat, height (Check for gender differences in each aspect).

-Ask for her perceptions of child’s height (probe for her Interpretations of short stature in a child, and determinants of height of a child; difference between ‘short stature and *kudumaa*: probe for markers of *kudumaa*)

-Probe for her opinion on the growth of a child who is fat (what makes a child to be fat?)

-Probe on her perception of child’s weight in relation to her/his growth (determinants of weight of a child?)

-Probe: What do you do to make [name] grow well? (Probe: traditional preventive measures, sleeping under a bed net, environment is clean, take to hospital for check-up, nutritious food etc). What more do you wish you could do?

-In case [Name] was mentioned to not grow well, ask: why do you think so? Please tell me about the growth experience of [Name]. (Probe on markers i.e. physical appearance, social interaction, weakness, not standing, inactive, not speaking, skin, hair, cheeks).

-What do you think made [Name] to not grow well?

-What did you do when you realized that there is growth issue in [*Name*]? (Probe for different actions e.g. go to health facilities, consulting traditional healers, use traditional herbs, -Ask what motivates her decisions?)

-(Please ask about growth status that was not the case in QN 4). Ask: do you know of a child that grew well? / did not grow well? Please tell me about her/him. How did he/she look like?

1. Are you aware of ‘utapiamlo’? Have you ever seen a child with Utapiamlo? How does s/he look like? (Probe on markers)

**Contextual factors for child growth**

1. As a mother, what are your responsibilities within the family? How does that influence growth of your children?
2. Who helps you with taking care of the children when you are (a) at home not busy (b) at home but busy (c)away from home for attending your daily responsibilities (d) the child is ill? - In each response probe for type of support received).
3. How does your husband/partner support you in taking care of [Name]? (Probe on types of support from husband/partner e.g. taking children to growth monitoring, hospitals, paying for school fees, feeding children, etc).
4. Please tell me something about the environments that underlie your ability to provide good care to [Name]. (Probe on: Husband’s / partner’s behavior, relationship with her husband or child’s father, access to resources, control over resources& family income, division of labor, child’s behaviors, environment, health system, food issues, community factors, income, health issues, beliefs, socio-cultural practices, marital status i.e. single, polygamous married etc).

-Can you think of a time that you wanted to take care of your child, but you could not? Please tell me about that?)

1. As a mother, what support do you need from your husband/partner, family, community, environment, health workers, policy makers) to make [Name] grow well?
2. What do you think of ‘kubemenda’? Has it ever happened to a child of any of people you know? Please tell me a story about it. (Probe on implication to couple’s relationship, community’s attitude? Who is generally blamed, in what way? What do you think about it?]
3. Do you know of a person that stays with a child who is not her/his own? What are the reasons that made that person to stay with the child? How do you consider the growth of that child? Why do you say so? (Probe on environment that the participant thinks influences growth of that child).
4. In this village, where do you get water for domestic use? (probe on multiple water sources, main and regular water source for drinking, How she prepares water for drinking, child feces disposal, place for hand-washing, use bush/field for open defecation, reliability and seasonality of water sources, time to collect water during wet and dry seasons).

**Child feeding practices**

1. How did you feed yourself when you were pregnant (probe on down-eating, ANC visits, advice given by ANC workers, food taboos, why?)
2. Kindly tell me about your child feeding practices. (Probe on the following:

-Breast feeding (initiation, colostrum, number of feeds a day, night feeding)

-How do you decide to give the child complementary food? (Probe on timing and reasons, including markers of growth, gender differences)

-What types of complementary food do you provide to your children (water, gripe water, juice, porridge, mashed potatoes, cow’s milk: probe on how cow’s milk is given, food taboos) (ask the participant to specify foods for various categories of under-fives)

-How many times children are fed in a day(ask this for various categories of under-five?)

- When do children start eating with the adults (age)

-Eating habits e.g. whether children and adults eat together in one bowl etc. (probe food distribution)

-Preparation methods and storage of foods

- When did you stop breastfeeding your children and why? (Probe on gender differences)

- Mother’s knowledge on recommendations in terms of breastfeeding when a mother is HIV positive?

**Experience with growth monitoring services**

1. What do you do when you realize that [N*ame*] is ill? (Probe for different actions e.g. go to health facilities, consulting traditional healers, use traditional herbs, -Ask what motivates her decisions? Check for availability of health facility in the area - check who is involved in child health care decisions?
2. Please tell me about growth monitoring services offered in your area (probe on a place where it is offered, who is providing the service, how often, if she takes her child to growth monitoring)
3. When was your last visit to the clinic? Kindly tell me what happened during this visit from the beginning to the time you left the facility).

-What did the health worker tell you about growth outcome of your child? (Weight improving / not). In case weight drop was not mentioned, Probe: during any of your visits, have you ever been told that your child’s weight has dropped / not increasing? How did you feel? What was your personal opinion on your child’s growth? What health information, advice provided? What did you do to improve weight of your child?)

1. Can you show me your youngest child's growth chart? What was your child’s weight at birth? How do you understand the chart? (colors, position in the chart) What do you think about this? (Probe on participant’s opinion on other criteria to be considered in assessing growth of under-five children? Note if both weight and height have been measured.
2. Have you ever missed any of your child’s growth monitoring clinics? What were the reasons? (Probe for participant’s view on the reasons that make mothers abandon attending growth monitoring clinics)
3. We are now approaching end of our discussion. What do you suggest to be done so as to improve growth monitoring activities?
