# Supplementary material for: 'He usually has what we call normal fevers’: Cultural perspectives on healthy child growth in rural Southeastern Tanzania: An ethnographic enquiry
Source: PLoS One. 2019 Sep 11;14(9):e0222231. doi: 10.1371/journal.pone.0222231 (PMC6738644; doi:10.1371/journal.pone.0222231)
Supplement: S6 File — (DOCX) [file pone.0222231.s006.docx]

**Appendix 5a: KII Guide: Health worker**

**Participants’ background information**

Age…………

Sex …………………..

Tribe ……………………

Religion …………………

Education level …………………..

Specialization ……………………..

Marital status………………………

**Opening questions**

1. Please tell me about yourself. How long have you lived in this village? How long have you worked as a health worker (Probe for specific roles as a health worker)?
2. How would you describe a young child? - as understood in your community? (age, not knowing things, helpless, does not know what is good and bad for him/her, preschool)
3. Where do people in this village get health services? (Probe for all sources of health)

**Perceptions towards optimal child growth**

1. Can you describe who is a healthy child as understood in your community? How about the unhealthy one? (Check if the informant has different opinion on how a healthy and unhealthy child looks).
2. In your community, who is considered to be a child who is growing well? (Probe: How can someone know that a child is growing well? (Dimensions: social interaction, weight, activeness, playfulness, eating habits, sleeping, emotional, cognitive, motor development, recognizing people, things, being fat, height; probe on how a child is expected to grow from when it was born to age (Check for gender differences).

-Ask for community’s & informant’s perceptions of child’s height (probe for Interpretations of short stature in a child, and determinants of height of a child; difference between ‘short stature and kudumaa: probe for markers of kudumaa)

-Probe for community’s & informant’s perception of the growth of a child who is fat (what makes a child to be fat?)

-Probe on perception of child’s weight in relation to her/his growth (determinants of weight of a child?)

1. How can a child who is not growing well be described? (Probe: How can someone recognize that a child is not growing well? (Probe on criteria used in recognizing poor growth – both growth faltering and over nutrition).
2. Please tell me something about the children that come to the child care clinic / health facility for growth monitoring. Generally, what can you say about their growth? Are they growing well? Why do you say so? [probe for markers]

**Contexts of child growth**

1. What are common growth problems among young children in your community?
2. What might be the factors that contribute to poor growth of young children in Malangali village? (Probe on: parents’ behavior, parents’ relationship, down-eating during pregnancy, access to resources, decision making over resources, family income & child health care; division of labor, child’s behaviors, environment, health system, food issues, community factors, income, health issues, beliefs, socio-cultural practices, marital status i.e. single, polygamous married etc).

-Do you know of any child/children who live in a disadvantaged environment? Please tell me a story about that. (Probe about the environment, quality of care to the child & participant’s opinion on growth of that particular child)

1. Please tell me something about ‘kubemenda’ – as understood in this community? (Probe: How does it happen? Signs? Remedies? How do the community members take it? Who is generally blamed, in what way? Any possible implication to couple’s relationship?

-Probe for informant’s opinion on kubemenda (myth or reality?).

-If s/he considers it to be a myth, what does s/he think it is the case?

-If s/he considers it to be a reality, please ask: Has it ever happened to a child of any of mothers who come for growth monitoring / people you know? Please tell me a story about it, and service provided to a child.

1. What support do you think parents/caregivers in this village need from family, community, health workers, policy makers etc so that their children grow well?

**Experience with health services / growth monitoring**

1. What do parents / caregivers in this village usually do when they think that there is a problem in growth of their children? (Probe: for different actions that caregivers took/take and reasons for that e.g. go to health facilities, consulting traditional healers, use traditional herbs, -what motivates their decisions?).

-Ask for narratives about any specific health / growth issues in children that necessitate the decisions to consult traditional healer or use of traditional medicine?

-Probe on self medication (reasons, impacts on child’s health)

-Knowledge about past & present interventions implemented in the village to promote child growth

1. Please tell me about growth monitoring services that you offer in your area (probe on the process of service provision, a place where it is offered, who is providing the service, how often, things that are checked for, charts used, opinions on mothers’ comprehension of the charts & other services provided during the clinic).
2. For many years now in child care programs growth of young children has been assessed by measuring their weight and sometimes height for age. What do you think about this?

-Have you had some experiences when you suspected problems while the measures were alright or other way? Please describe that case / experience.

-What other issues would you suggest to be considered in assessing growth of young children? (For each response, ask why?)

1. In growth monitoring practice, when you identify a child with growth faltering, what do you usually do? Please tell me about the last case of growth faltering and all things that happened regarding that child? What was caregivers’ reaction on the feedback regarding growth outcome of her child?

-Probe about challenges that the informant face in providing growth monitoring service.

1. Elsewhere, the experience shows that majority of mothers abandon attending growth monitoring clinics during the child’s first or second year of age. What is the experience in this community? What do you think might be the reasons for this?
2. We are now approaching end of our discussion. Do you have anything to add on child growth and growth monitoring?
